# MEM: An Algorithm for the Reliable Detection of Microsatellite Instability (MSI) on a Small NGS Panel in Colorectal Cancer

**DOI:** 10.3390/cancers13164203

**Published:** 2021-08-20

**Authors:** Guillaume Herbreteau, Fabrice Airaud, Elise Pierre-Noël, Audrey Vallée, Stéphane Bézieau, Sandrine Théoleyre, Hélène Blons, Simon Garinet, Marc Guillaume Denis

**Affiliations:** 1 Laboratoire de Biochimie et Plateforme de Génétique Moléculaire des Cancers, CHU Nantes (Nantes University Hospital), 44093 Nantes, France; elise.pierrenoel@chu-nantes.fr (E.P.-N.); audrey.vallee@chu-nantes.fr (A.V.); sandrine.charpentier@chu-nantes.fr (S.T.); marc.denis@chu-nantes.fr (M.G.D.); 2 Service de Génétique Médicale, CHU Nantes (Nantes University Hospital), 44093 Nantes, France; fabrice.airaud@chu-nantes.fr (F.A.); stephane.bezieau@chu-nantes.fr (S.B.); 3 Plateforme de Génétique Moléculaire des Cancers, Hôpital Européen Georges Pompidou, Assistance Publique des Hôpitaux de Paris (Greater Paris University Hospitals), 75015 Paris, France; helene.blons@aphp.fr (H.B.); simon.garinet@aphp.fr (S.G.)

**Keywords:** microsatellite instability, deficient mismatch repair system, colorectal cancer, NGS, expectation-maximisation algorithm

## Abstract

**Simple Summary:**

Microsatellite instability (MSI) assessment has become a major issue in the management of colorectal cancer, with the recent approval of anti-PD1 immunotherapies in MSI-metastatic colorectal cancer. The reference PCR method (MSI-PCR) can be costly, time and tissue-consuming. However, NGS could facilitate the assessment of MSI status while simultaneously screening for targetable oncogenic mutations (KRAS, NRAS, BRAF) for any colorectal cancer, but the algorithms developed to date use a large number of microsatellites that have not been approved by international guidelines and which are generally incompatible with small NGS panels. We present the MEM algorithm, which mimics the interpretation of MSI-PCR data by a human operator to reliably assess MSI status using only five validated microsatellites (BAT-25, BAT-26, NR-21, NR-24 and NR-27). We demonstrated that the MEM algorithm was in perfect agreement with MSI-PCR results, in terms of both MSI status and individual microsatellite status, in a cohort of 146 patients.

**Abstract:**

Purpose: MEM is an NGS algorithm that uses Expectation-Maximisation to detect the presence of unstable alleles from the NGS sequences of five microsatellites (BAT-25, BAT-26, NR-21, NR-24 and NR-27). The purpose of this study was to compare the MEM algorithm with a reference PCR method (MSI-PCR) and MisMatch Repair protein immunohistochemistry (MMR-IHC). Methods: FFPE colorectal cancer samples from 146 patients were analysed in parallel by MSI-PCR and NGS using the MEM algorithm. MMR-IHC results were available for 133 samples. Serial dilutions of an MSI positive control were performed to estimate the limit of detection. Results: the MEM algorithm was able to detect unstable alleles of each microsatellite with up to a 5% allelic fraction. Of the 146 samples, 28 (19.2%) were MSI in MSI-PCR. MEM algorithm results were in perfect agreement with those of MSI-PCR, at both MSI status and individual microsatellite level (Cohen’s kappa = 1). A high level of agreement was noted between MSI-PCR/MEM algorithm results and MMR-IHC results (Cohen’s kappa = 0.931). Conclusion: the MEM algorithm can determine the MSI status of colorectal cancer samples on a small NGS panel, using only five microsatellites approved by international guidelines, and can be combined with screening for targetable mutations.

## 1. Introduction

Microsatellite instability (MSI) is the molecular consequence of a deficient mismatch repair system (dMMR) [1]. Microsatellites are DNA sequences formed by the continuous repetition of patterns comprising 1 to 6 nucleotides. During replication, the number of repetitions (and therefore the length) of a microsatellite may vary due to slipped strand mispairing. The mismatch repair system (MMR) is involved in repairing these replication slippage errors, notably for mono-nucleotide microsatellites [2]. dMMR triggers a variation in the length of microsatellites and genetic instability associated with the onset of many cancers: Lynch syndrome, a key cancer predisposition, is linked to constitutional dMMR, while sporadic dMMR is found in 15% to 20% of colorectal and gastric cancers, in 20% to 30% of endometrial cancers and most solid cancers are likely to exhibit sporadic dMMR at lower frequencies [3,4].

dMMR is usually demonstrated by identifying the loss of expression of at least one of the MMR proteins (MLH1, MSH2, MSH6 or PMS2) by immunohistochemistry (MMR-IHC). dMMR can also be demonstrated by revealing its consequence, microsatellite instability (MSI), using molecular biology methods (MSI-PCR) [5,6]. MSI-PCR requires the amplification of a locus comprising a microsatellite of interest by PCR and then analyses the length of the amplicons generated, usually by electrophoresis of the amplification products on a capillary sequencer [7]. The analysis of five poly-A mononucleotide microsatellites (BAT-25, BAT-26, NR-21, NR-24 and NR-27; Supplementary Table S1) are recommended by the revised Bethesda guidelines and the ESMO guidelines for mCRC MMR status determination [5,6]. The MSI phenotype is defined by the instability of at least two of the five microsatellites, while the stability of microsatellites (MSS) is defined by the instability of zero or one microsatellite [6].

Several studies have recently shown that dMMR is predictive of a good response to immune checkpoints inhibitors in several cancer types. Indeed, genetic instability induced by dMMR is likely to generate an increased number of neo-antigens, which can confer better tumour immunogenicity [8]. Pembrolizumab, an anti-PD1 immunotherapy, showed superior efficacy to chemotherapy as a first-line treatment for dMMR metastatic colorectal cancer (dMMR-mCRC) in the phase III KEYNOTE-177 trial [9]. In addition, pembrolizumab has proved effective as a second-line treatment for other types of dMMR cancers (mainly endometrial, gastric, cholangiocarcinoma and pancreatic cancers) in the Phase II KEYNOTE-158 trial [10]. In 2017, the US Food and Drug Administration (FDA) granted accelerated approval to pembrolizumab for the first-line treatment of dMMR-mCRC and as a second-line treatment for any unresectable or metastatic dMMR solid cancer when no other treatment options were available [11]. Another anti-PD1 immunotherapy, nivolumab, has also proved effective alone or in combination with ipilimumab (anti-CTLA4 immunotherapy) as a second-line treatment for dMMR-mCRC in the Phase II CheckMate-142 trial [12,13]. Nivolumab and nivolumab-ipilimumab combination therapy have been approved by the FDA as second-line treatments for dMMR-mCRC, and a phase III trial is currently underway (CheckMate-8HW [14]).

With the development of these new therapeutic options, the determination of MMR status has become a critical point in cancer management. However, costs, time and tissue consumption relating to MMR-IHC and MSI-PCR analyses may represent limiting factors, particularly with the pembrolizumab site-agnostic indication. In addition, MSI-PCR and MMR-IHC are rarely performed on tumours with low dMMR prevalence (<1%), although the impact on treatment could be significant. The determination of MSI status by Next-Generation Sequencing (MSI-NGS) seems an alternative of choice since it allows simultaneous high-throughput analysis of numerous samples and can be coupled with the search for somatic mutations of theranostic interest, which is already carried out in routine practice [15]. Many MSI-NGS algorithms have been developed [16,17,18,19,20,21,22,23,24,25,26,27,28]; however, they require sequencing of large panels of microsatellites that have not been approved by international guidelines, and their diagnostic performance is often imperfect.

In this context, we present a novel MSI-NGS algorithm called MEM (MSI assessment by Expectation-Maximisation algorithm) to determine MSI status by analysing only the NGS sequencing data of the five microsatellites validated by the Bethesda and ESMO international guidelines (BAT-25, BAT-26, NR-21, NR-24 and NR-27), using a method that mimics the interpretation of MSI-PCR data by a human operator.

## 2. Materials and Methods

### 2.1. MEM Algorithm

In MSI-PCR, a microsatellite is considered stable if its length distribution is comparable to the reference distribution and unstable if its length distribution corresponds to a mixture model, i.e., the mixture of several sub-distributions of different mean lengths (generally a sub-distribution corresponding to the length of the stable allele, similar to the reference distribution, and one or more sub-distributions of different mean lengths, corresponding to the unstable alleles).

**Figure 1:**
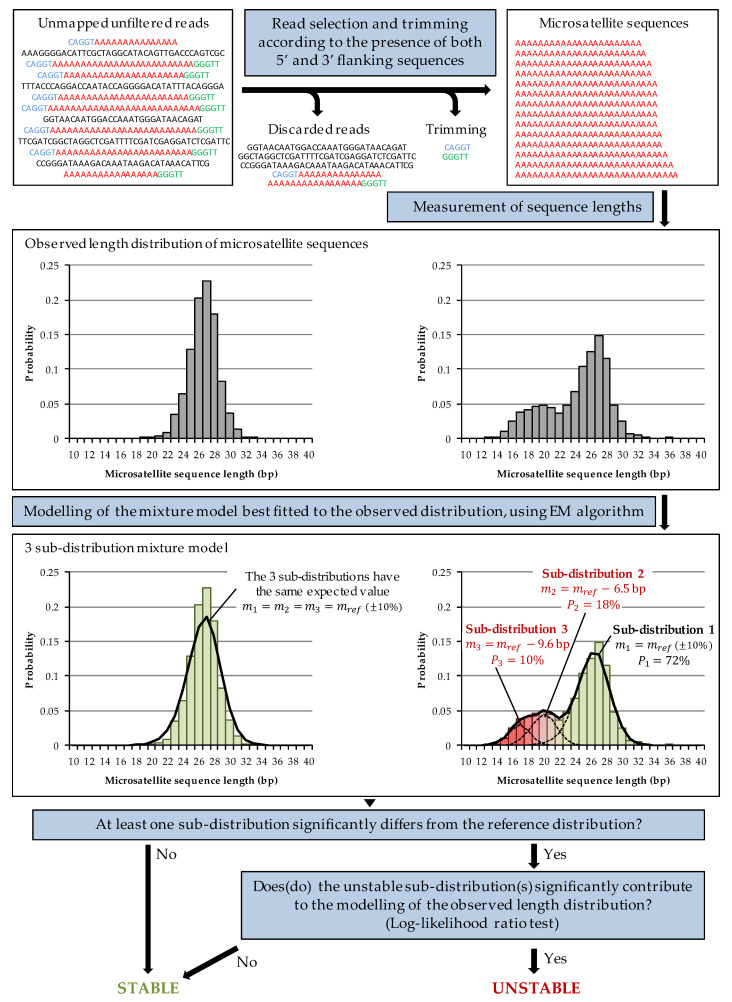
MEM workflow summary. Microsatellite sequences are obtained by trimming its 5′ and 3′ flanking sequences in unmapped reads when both are identified using Smith–Waterman alignment (see Supplementary Table S1 for parameters). The lengths of the microsatellite sequences are measured to determine their statistical distribution (left panel: example of a BAT-26 stable distribution; right panel: example of a BAT-26 unstable distribution). MEM uses the EM algorithm to build a three sub-distribution mixture model of the observed distribution. If at least one sub-distribution differs significantly from the reference distribution and contributes significantly to the modelling according to a log-likelihood ratio test, then the microsatellite is considered unstable; otherwise it is considered stable.

MEM is a Java-based bioinformatics algorithm built on CLC Genomics Workbench 20 (QIAGEN, Hilden, Germany), available at https://github.com/MGPC-Nantes/MEM (accessed on 19 August 2021). MEM attempts to closely replicate the MSI-PCR interpretation method. Since the amplification steps during NGS library preparation induce replication slippage of microsatellite sequences as with MSI-PCR, MEM identifies the stable or unstable nature of each microsatellite by (i) determining the length distribution of microsatellite sequences, without post-analytical bias related to filtering or alignment of the sequencing data, and (ii) determining whether the observed distribution corresponds to a mixture model whose sub-distributions differ from the reference distribution (Figure 1; see Appendix A for a detailed description).

The first step of the MEM analysis is to identify, for each microsatellite, the 5′ and 3′ flanking sequences of the microsatellite from unmapped and quality unfiltered, paired-end reads using Smith–Waterman alignment (Supplementary Table S1). If both 5′ and 3′ flanking sequences are identified in a read, then that read is trimmed from these sequences, and the resulting sequence is retained if it contains a homopolymeric sequence (otherwise, the read is excluded from the analysis). The microsatellite length distribution is then determined by measuring the length of each trimmed sequence.

The reference length distribution of each microsatellite was determined with the same method, using unaligned reads from merged FASTQ of 36 MSS samples showing the stability of all five microsatellites in MSI-PCR and expressing MLH1, MSH2, MSH6 and PMS2 in MMR-IHC.

To determine whether a microsatellite is unstable, MEM builds a model of the observed length distribution using a three sub-distribution mixture model (assuming one sub-distribution for the stable allele and up to two sub-distributions for two unstable alleles). The “shape” of each sub-distribution n is extrapolated from the reference length distribution previously defined for the microsatellite on MSS samples, and each sub-distribution is defined by two parameters: its expected value $m_{n}$ and the proportion $P_{n}$ of the mixture model represented by that sub-distribution. MEM uses the Expectation-Maximisation algorithm to determine a vector of parameters $\left\{m_{1},m_{2},m_{3},P_{1},P_{2},P_{3}\right\}$, defining a mixture model approximating the observed length distribution with maximum likelihood. If the expected value $m_{n}$ of a sub-distribution n is equal to the mean of the reference distribution, plus or minus 10%, then this sub-distribution is considered to be associated with a stable allele; otherwise, it is considered to be potentially associated with an unstable allele.

If all the sub-distributions are stable, or if the unstable sub-distributions represent less than 2% of the mixture model, then the microsatellite is considered stable. If there are one or more unstable sub-distributions representing more than 2% of the mixture model, then MEM compares the log-likelihood of the full mixture model to the log-likelihood of a mixture model, including only the stable sub-distributions, using a log-likelihood ratio test. If the complete model represents the observed length distribution significantly better than the model, including only the stable sub-distributions, then the microsatellite is considered unstable, and MEM quantifies the proportion of unstable alleles and their mean lengths from the parameters of the mixture model. Otherwise, MEM estimates the power of the log-likelihood ratio test for the number of sequences in the microsatellite: if the power of the test is greater than 80%, then the microsatellite is considered stable; otherwise the analysis of this microsatellite is considered to be non-contributory. Similar to MSI-PCR, the sample is then considered MSI if at least two out of five microsatellites are unstable.

### 2.2. Evaluation of the Limit of Detection

In order to evaluate MEM performance for the detection of instabilities in the presence of a small percentage of unstable alleles, DNA samples were reconstituted by diluting DNA from an MSI positive control with a high proportion of unstable alleles for the five microsatellites in MSI-PCR and the DNA from an MSI negative control. Six dilutions were prepared (1/4, 1/8, 1/12, 1/16, 1/20), then sequenced by NGS according to the protocol described below and analysed with the MEM algorithm.

### 2.3. Validation on Tumour Samples

NGS data from colorectal cancer samples sent to Nantes University Hospital between 1 January and 31 December 2019 for MSI assessment by MSI-PCR in conjunction with testing for KRAS, NRAS and BRAF by NGS were re-analysed retrospectively with the MEM algorithm.

The DNA of formalin-fixed paraffin-embedded (FFPE) colorectal cancer samples was extracted using the Maxwell^®^ RSC RNA FFPE Kit. NGS libraries were produced from these DNA extracts using the QIAseq Targeted DNA Custom Panel (QIAGEN, Hilden, Germany) kit, an amplicon library construction kit based on Anchored Multiplex PCR (AMP) technology. This panel targeted, among others, KRAS, NRAS and BRAF genes and BAT-25, BAT-26, NR-21, NR-24 and NR-27 microsatellites (see Supplementary Table S1 for the genomic positions of the primers targeting microsatellites). NGS libraries were prepared according to the supplier’s recommendations and then sequenced on a MiSeq sequencer (Illumina, San Diego, CA, USA).

The MSI-PCR analysis was carried out by the genetics laboratory of Nantes University Hospital using an in-house kit targeting BAT-25, BAT-26, NR-21, NR-22 and NR-24 microsatellites (see [29] for the list of primer sequences). Fragment analysis was performed on a 3500 xL Genetic Analyser capillary sequencer (Applied Biosystems, Waltham, MA, USA) and interpreted in comparison with a control sample stable for the five microsatellites, on GeneMapper software 5, by trained operators who were kept blinded to MEM results.

Both MEM algorithm results and MSI-PCR results were collected in an anonymized computer database together with the percentage of cancer cells in the sample, estimated by examining hematoxylin and eosin-stained tissue slides and the tumour expression of MLH1, MSH2, MSH6 and PMS2 evaluated by MMR-IHC, provided by the pathologist.

### 2.4. Ethical Aspects

No result related to MSI assessment by MEM was communicated for the purpose of patient management. The MSI-PCR or MMR-IHC results were not rechecked against MEM results. According to French and European legislation, the use of anonymous data does not require ethics committee approval. This study has been registered at Nantes Hospital by the Local Data Protection Officer under reference TS005-BIO.2019_4.

## 3. Results

### 3.1. Limit of Detection

The MSI positive control used to assess the limit of detection showed 73%, 77%, 74%, 70% and 77% of unstable alleles for BAT-25, BAT-26, NR-21, NR-24 and NR-27, respectively, according to the MEM analysis. Among the samples prepared by diluting this MSI positive control with an MSS negative control, MEM identified MSI status for 1/4, 1/8, 1/12 and 1/16 dilutions, i.e., a limit of detection of approximately 5% of unstable alleles. For all microsatellites, the proportions of unstable alleles quantified by MEM were consistent with expected proportions, taking into account the dilution factors and the initial proportion of unstable alleles in the positive control (R^2^ > 0.99 for each microsatellite, data not shown).

### 3.2. MEM Algorithm vs. MSI-PCR Comparison

**Figure 2:**
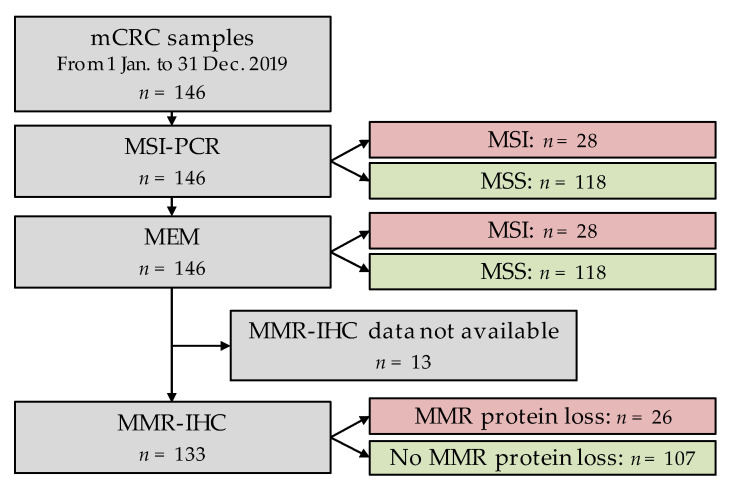
Study workflow.

A total of 146 colorectal cancer samples were sent to Nantes University Hospital between 1 January and 31 December 2019, to test for KRAS, NRAS or BRAF mutations by NGS and microsatellite instability by MSI-PCR. A total of 28 samples out of 146 (19.2%) were MSI according to MSI-PCR, and the remaining 118 samples were MSS (Figure 2). The percentage of cancer cells in samples exceeded 50% for 117 samples (80.1%), ranged from 25% to 50% for 28 samples (19.2%) and from 10% to 25% for a single sample (0.7%). No sample contained less than 10% cancer cells.

NGS data obtained from these samples were re-analysed with the MEM algorithm. MEM conclusions were entirely consistent with those of MSI-PCR (Cohen’s kappa = 1; Table 1). All MSI samples in MSI-PCR were identified as MSI by MEM (28/28), and all MSS samples in MSI-PCR were identified as MSS with MEM (118/118). Considering MSI-PCR as gold-standard, MEM had sensitivity, specificity and positive and negative predictive values of 100% for the determination of MSI status.

In terms of each individual microsatellite, the MEM algorithm was also entirely consistent with the MSI-PCR results for BAT-25, BAT-26, NR-21 and NR-24 (Cohen’s kappa = 1 in each case; Table 2). For a single MSI sample for which BAT-25, BAT-26, NR-21 and NR-22 were unstable with the MEM algorithm, MSI-PCR did not generate enough amplicons of the BAT-26 locus to allow interpretation (*n =* 145 for BAT-26).

Concordance between MEM and MSI-PCR could not be established for NR-27 because the MSI-PCR analysis used did not evaluate this microsatellite. However, MEM results for NR-27 were strongly concordant with MSI status in MSI-PCR (Table 3). Among the 28 MSI samples, MEM identified NR-27 as unstable for 26 samples (92.9%), stable for 1 sample and non-contributory for 1 sample due to an overall constitutive lack of NR-27 coverage, probably due to deletion of the NR-27 locus (as this microsatellite was targeted by several primers in our panel). Among the 118 MSS samples, NR-27 was identified as stable in 116 cases (98.3%). NR-27 was unstable for 2 MSS samples, with both cases having an unstable allele with an average length of −4 bp compared to the reference distribution and an allele frequency of approximately 50% according to MEM, raising suspicions of NR-27 polymorphism in these samples.

### 3.3. MEM vs. MMR-IHC Comparison

MMR-IHC data were available for 133 samples, including 107/118 MSS samples and 26/28 MSI samples in MSI-PCR.

All of these 26 MSI samples (100%) displayed loss of expression of at least one of the MMR proteins (MLH1, MSH2, MSH6 or PMS2). Among the 107 MSS samples, 104 showed no loss of expression of MMR proteins in MMR-IHC (96.3%; Table 4). As the results obtained with MEM were entirely consistent with those of MSI-PCR, the same comparison was observed with our algorithm, with three samples showing MSS status with MEM and loss of expression of at least one MMR protein in MMR-IHC. For the three samples, the percentage of cancer cells was greater than 25%, all the microsatellites analysed were stable, and the loss of expression in MMR-IHC affected the MLH1 and PMS2 proteins, a situation typically observed in the case of sporadic dMMR.

## 4. Discussion

MEM is the first MSI-NGS algorithm using only five microsatellites validated by international guidelines for colorectal cancer management (Bethesda revised, ESMO). MEM is compatible with small NGS panels and attempts to faithfully reproduce the principles of MSI-PCR, both in the establishment of microsatellite length distributions and their interpretation. Because of the closeness between the two methods, MEM produces results that are fully consistent with MSI-PCR for the analysis of colorectal cancer samples at both MSI status and individual microsatellite levels.

Several MSI-NGS algorithms have been developed to assess MSI status from NGS data (mSINGS/MSIplus [16,17], MANTIS [18], MSIseq [19], MSIsensor [20], MSI-ColonCore [21], ELMSI [24], MSICare [25], mSILICO [26], NovoPM-MSI [27], USCI-MSI [28], Cortes-Ciriano et al. [22], Lu et al. [23]). Similar to MEM, these algorithms roughly operate according to two main steps: firstly, they determine the length distribution of microsatellite sequences, and secondly, they compare the observed distribution to a reference distribution to determine the stable or unstable status of the microsatellite in a standardised and reproducible manner. These algorithms differ essentially in the way in which both steps are performed.

Most algorithms identify and determine the length of microsatellite sequences by recognising the sequences flanking the microsatellite, on aligned reads in BAM format (mSINGS/MSIplus [16,17], MANTIS [18], ELMSI [24], Cortes-Ciriano et al. [22]), while some algorithms are based on the analysis of indel variants identified in microsatellite sequences (MSIseq [19], Lu et al. [23]) or determine the length distribution of microsatellite sequences by alignment on all possible length variants of the microsatellite (MSIsensor [20], MSI-ColonCore [21]). The MEM algorithm determines the length distribution of the microsatellites by recognising the flanking sequences directly on unaligned, unfiltered, paired-end reads in FASTQ format. This method seeks to replicate as far as possible the MSI-PCR approach in which the amplicons generated are selected by primer choice, regardless of the internal sequence of the amplified locus. In this way, MEM maximises the number of sequences obtained for each microsatellite and essentially limits the introduction of bias in the length distribution related to the exclusion of some reads with low sequencing quality (as is often the case for homopolymeric sequences) or where marked instability would interfere with mapping by rendering their sequence too different from the reference sequence.

MSI-NGS algorithms also differ in terms of the methods used to interpret the length distribution of microsatellite sequences. Some algorithms use statistical methods to compare the distribution, such as the Kolmogorov–Smirnov test (Cortes-Ciriano et al. [22]), Chi-squared test (MSIsensor [20]) or scores approaching it (MANTIS [18]). Other algorithms analyse parameters related to distribution dispersion, such as the number of different lengths observed at a significant proportion in microsatellite sequences (mSINGS/MSIplus [16,17]) or the proportion of microsatellite sequences whose length is within a reference interval (MSI-ColonCore [21]), or parameters related to distribution skewness (mSILICO [26]). Finally, some algorithms use interpretation methods based on machine learning algorithms (MSIseq [19]). For its part, the MEM algorithm replicates the MSI-PCR interpretation method by a human operator, evaluating whether the observed length distribution of a microsatellite is deemed a mixture model, using an Expectation-Maximisation algorithm and empirical reference length distributions obtained on a cohort of MSS samples. ELMSI, a recently published algorithm, also uses an Expectation-Maximisation algorithm to determine the stability of microsatellites over large panels but in our experience, the normal distribution used to characterise the reference distribution of each microsatellite does not match the distributions actually observed [24].

Although most MSI-NGS algorithms can determine the MSI status of a cancer sample with reported good sensitivity and specificity (Table 5) [16,18,19,20,21,22,23,24], they may be unreliable for determining the stable or unstable status of each microsatellite individually. These methods manage to compensate for this shortcoming by simultaneously analysing several tens to several thousands of microsatellites, combined with comparison to a matched non-tumour sample for certain algorithms, thus reducing the risk and impact of microsatellite misclassification. However, analysing this number of microsatellites requires the use of large NGS panels, culminating in whole-exome sequencing, which is problematic since it increases analytical costs and diminishes multiplexing capacity. On the other hand, the use of microsatellite panels not validated by international recommendations may be a limiting factor in terms of therapeutic indications for immunotherapy.

The MEM algorithm analyses each microsatellite individually in a standardised manner and is highly consistent with MSI-PCR results. Indeed, in this study, we have shown perfect agreement between MSI-PCR results and MEM results for BAT-25, BAT-26, NR-21 and NR-24, while NR-27, which was not evaluated in MSI-PCR, demonstrated excellent agreement with the MSI status of samples, with performance levels similar to those previously described for this panel of microsatellites in MSI-PCR (sensitivity and specificity: 93.9% and 99.0%, respectively, in MSI-PCR according to Goel et al., vs. 92.9% and 98.3%, respectively, with MEM, in our study [7]). Given this level of reliability for each individual microsatellite, MEM can be used to determine the MSI status of colorectal cancer samples by analysing only the microsatellites validated by international recommendations. In addition, the reduced number of microsatellites analysed and the non-requirement of a matched non-tumour sample considerably limits the size of the NGS panel to be used, limits the run-time (less than 10 s per sample for the whole process) and maintains a large multiplexing capacity, compatible with the number of samples to be analysed assuming a site-agnostic indication for immunotherapy.

One of the main constraints associated with this algorithm relates to the need for sufficient coverage of each microsatellite in order to obtain homogeneous length distribution. According to the quality parameters used in our study, a minimum of 689, 958, 707, 579 and 746 reads were required to allow the interpretation of BAT-25, BAT-26, NR-21, NR-24 and NR-27 length distributions, respectively. Optimisation of the NGS panel used may be necessary to guarantee sufficient coverage.

Given the proximity between MEM and MSI-PCR, both analyses also have similar limits and continue to complement MMR-IHC. Although MSI-NGS can be coupled to the sequencing of MMR genes to identify mutations associated with Lynch syndrome, neither MSI-NGS nor MSI-PCR can identify the loss of expression of an MMR protein: MMR-IHC, possibly associated with the search for MLH1 promoter hypermethylation, remains essential in order to distinguish sporadic dMMR from Lynch syndrome [6]. In addition, both MSI-PCR and MSI-NGS can produce false-negative results that MMR-IHC can help to identify: in our study, we identified three potential cases in which loss of expression of MMR proteins was observed in MMR-IHC despite MSS status with both MSI analyses. Several factors can limit the sensitivity of MSI-PCR and, by extension, the sensitivity of MSI-NGS: on the one hand, the detection of an unstable microsatellite is conditioned by the proportion of unstable alleles in the sample and can therefore be limited for samples with a small proportion of cancer cells. However, this limitation was not encountered in our study, since almost all of the samples (the three potential false-negatives in particular) presented tumour cellularity greater than 25%, while MEM showed an ability to identify microsatellite instability at allelic frequencies as low as 5%. Conversely, the sensitivity of MSI-PCR and MSI-NGS is also determined by the degree of instability: the lower the variation in length between an unstable microsatellite and the wild-type allele, the harder the identification process. If this phenomenon does not interfere to any considerable extent with the analysis of colorectal cancer samples, which are generally unstable to a significant degree, it is more frequently described in other tumour types, such as endometrial cancers [4,30]. As our study assessed the performance levels of the MEM algorithm only in evaluating the MSI status of colorectal cancers, a new assessment seems necessary to determine its analytical performance in other tumour types. In addition, some authors suggest that the analysis of only five microsatellites could be insufficient to identify all of the MSI samples, particularly for non-colorectal cancers for which the Bethesda panel microsatellites are not validated [3,4]. If the Bethesda panel is extended to incorporate new microsatellites or if new microsatellite panels adapted for MSI status determination are defined in some non-colorectal cancers, MEM could be adapted by updating the NGS panel and defining reference distributions for these new microsatellites on MSS control samples.

Conversely, MSI-NGS and MSI-PCR can be used to identify certain false-negatives of MMR-IHC, in particular when dMMR is linked to a loss of function of one of the MMR proteins without loss of expression. Both MSI-NGS and MSI-PCR have excellent specificity, with no false positives provided that care is taken to recognise potential polymorphisms of microsatellites. Although parallel analysis of a non-tumour sample is not required for the assessment of colorectal cancer, it should be carried out whenever the presence of a microsatellite polymorphism is suspected and particularly when the status of this microsatellite conditions the MSI status of the sample [31].

The MEM algorithm has been used since April 2020 at Nantes University Hospital for microsatellite instability assessment using NGS, thereby replacing MSI-PCR. From April 2020 to March 2021, 292 colorectal cancer samples were analysed: 36/292 were MSI (12.3%) with MEM. MMR-IHC was reported in 256 samples, including 34/36 MEM-MSI samples: all of these MEM-MSI samples demonstrated loss of expression of at least one MMR protein in MMR-IHC. Of the remaining 222 MEM-MSS samples, 221/222 had no loss of MMR protein expression, and 1 had a loss of expression of MLH1 and PMS2. Results were returned in conjunction with KRAS/NRAS/BRAF status within a median of 6 working days after receiving the samples (Q1–Q3: 6–7 days).

## 5. Conclusions

The MEM algorithm allows systematic determination of MSI status, analysing only the five microsatellites validated by international guidelines. MEM can be combined with the test for theranostic mutations on small NGS panels for all colorectal cancer samples. Further studies are needed to compare the MEM performance with other MSI-NGS algorithms and to evaluate it for other cancer types.

## Figures and Tables

**Table 1:** Concordance between MSI-PCR results and MEM results for MSI status.

		**MSI-PCR**		**Cohen’s Kappa**
		MSI	MSS	
**MEM**	MSI	28	0	1
	MSS	0	118	

**Table 2:** Concordance between MSI-PCR and MEM results for BAT-25, BAT-26, NR-21 and NR-24.

			MSI-PCR		Cohen’s Kappa
			Unstable	Stable	
**BAT-25**	**MEM**	Unstable	28	0	1
		Stable	0	118	
**BAT-26**	**MEM**	Unstable	27	0	1
		Stable	0	118	
**NR-21**	**MEM**	Unstable	28	0	1
		Stable	0	118	
**NR-24**	**MEM**	Unstable	26	0	1
		Stable	0	120	

**Table 3:** Concordance between MEM results for NR-27 and MSI status in MSI-PCR.

			MSI-PCR	
			MSI	MSS
**NR-27**	**MEM**	Unstable	26	2
		Stable	1	116

**Table 4:** Concordance between MEM results for MSI status and MMR-IHC.

		MMR-IHC		Cohen’s Kappa
		Loss of Expression of ≥1 MMR Protein	No Loss of Expression	
**MEM**	MSI	26	0	0.931
	MSS	3	104	

**Table 5:** Reported diagnostic performances for the main MSI-NGS algorithms for the determination of MSI status, considering MSI-PCR as gold-standard.

MSI-NGS Algorithm	Normal Sample Required	Microsatellite Count	Validation Cohort	Sensitivity	Specificity
mSINGS/ MSIplus	No	N/A (TGS)	Zhao [27]: *n =* 113	88.9%	99.0%
		15 to 2957	Salipante [16]: *n =* 108	96.4–100%	97.2–100%
		2539	Kautto [18]: *n =* 275	76.1%	99.7%
		3154	Lee [26]: *n =* 117	92.5%	100%
		230	Lee [26]: *n =* 117	95.0%	100%
		23	Lee [26]: *n =* 117	95.0%	93.7%
		11	Hempelmann [17]: *n =* 81	97.1%	100%
MSIsensor	Yes	N/A (WES)	Niu [20]: *n =* 242	98.6%	98.2%
		2539	Kautto [18]: *n =* 275	96.5%	98.7%
		N/A (WES)	Ratovmanana [25]: *n =* 333	85.5–90.2%	95.5–100%
		441	Ratovmanana [25]: *n =* 152	97.1%	73.3%
MANTIS	Yes	2539	Kautto [18]: *n =* 275	97.2%	99.7%
		N/A (TGS)	Zhao [27]: *n =* 113	88.9%	86.5%
MSI-ColonCore	Yes	90	Zhu [21]: *n =* 91	97.9%	100%
ELMSI	No	20 to 100	Wang [24]: simulated data	70.0–82.1%	N/A
MSICare	Yes	N/A (WES)	Ratovmanana [25]: *n =* 333	96.1–100%	97.0–100%
		441	Ratovmanana [25]: *n =* 152	99.3%	100%
mSILICO	No	3154	Lee [26]: *n =* 117	100%	100%
		230	Lee [26]: *n =* 117	100%	77.2%
		23	Lee [26]: *n =* 117	95.0%	100%
NovoPM-MSI	Yes	19	Zhao [27]: *n =* 113	88.9%	97.1%
USCI-MSI	Yes	9	Zheng [28]: *n =* 64	100%	100%
MEM	No	5	(present study): *n =* 146	100%	100%

N/A: Not Available; TGS: Targeted-Gene Sequencing; WES: Whole-Exome Sequencing.

## Data Availability

The data presented in this study are available on request from the corresponding author.

## Supplementary Materials

The following are available online at https://www.mdpi.com/article/10.3390/cancers13164203/s1, Table S1. Monomorphic microsatellites used by MEM for the determination of MSI status, genomic position of QIAseq AMP primers and MEM trimming parameters.

## Appendix A. MEM Algorithm

### Appendix A.1. Determination of the Length Distribution of Microsatellite Sequences

MEM is a bioinformatics algorithm built on CLC Genomics Workbench 20 (QIAGEN, Hilden, Germany). This algorithm directly analyses unaligned reads in FASTQ format (preventing mapping from triggering a bias in length distribution by excluding reads in which the microsatellite would differ substantially from the reference sequence, especially in cases of marked instability).

The first step of the MEM analysis consists in identifying, for each microsatellite, the 5′ and 3′ flanking sequences of the microsatellite among the unmapped and quality-unfiltered, paired-end reads, using Smith–Waterman alignment (See Supplementary Table S1 for the list of sequences used and the alignment parameters). If both 5′ and 3′ flanking sequences are identified within a read, then this read is trimmed from these sequences, and the resulting sequence is retained if it contains a homopolymeric sequence. Otherwise, if at least one of the flanking sequences is not identified or if the resulting trimming sequence does not contain any homopolymer, then the read is excluded from the analysis. The resulting trimmed sequences essentially correspond to the sequences of the microsatellite of interest: their length is measured, and the probability of each length is then determined by reporting the number of trimmed sequences presenting this length in relation to the total number of trimmed sequences obtained to establish the length distribution of the microsatellite.

(A1) $$p_{i}=\frac{N_{i}}{\sum_{i=0}^{L_{max}}N_{i}}$$

$p_{i}$: probability of occurrence of a microsatellite sequence of length i
$N_{i}$: number of trimmed sequences of length i
$L_{max}$: maximum length

The reference length distribution of each microsatellite was determined according to the method described, using unaligned reads from merged NGS data from 36 MSS samples with the stability of the five microsatellites in MSI-PCR and expressing MLH1, MSH2, MSH6 and PMS2 in MMR-IHC.

### Appendix A.2. Modelling of Length Distribution Using a Mixture Model

To determine whether a microsatellite is unstable, MEM determines a mixture model with three sub-distributions (assuming one sub-distribution for the stable allele and two for two unstable alleles) representing the observed length distribution with the maximum likelihood. This mixing model is defined by a vector θ of five independent parameters: $m_{1}$, $m_{2}$ and $m_{3}$, the expected value of each sub-distribution, and $P_{1}$ and $P_{2}$, the proportions of sub-distributions one and two, respectively, within the mixture model (given that proportion $P_{3}$ of the third sub-distribution is not an independent parameter, since $P_{1}+P_{2}+P_{3}=1$).

To create this type of mixture model, a continuous density function has to be defined for each sub-distribution, the expected value *m* of which can be parameterised. To this end, the shape of each sub-distribution is considered similar to the reference distribution. Thus, a continuous density function f(x,m) is extrapolated from this empirical discrete reference distribution using Parzen–Rosenblatt kernel density estimation:

(A2) $$f\left(x,m\right)=\frac{1}{h}\overset{L_{max}}{\underset{i=0}{\sum }}\left[K\left(\frac{S\left(x,m\right)-i}{h}\right)\times p_{i}\right]$$

h: a smoothing parameter called “bandwidth”. MEM uses a bandwidth h = 1

K(x): the kernel function. MEM uses the density function of the standard normal distribution as the kernel function, such as:

(A3) $$K\left(x\right)=\frac{1}{\sqrt{2}}e^{-\frac{1}{2}x^{2}}$$

S(x,m): a scaling function allowing a switch from a reference distribution with an expected value, $m_{ref}$, to distribution with an expected value, m, such as:

(A4) $$S\left(x,m\right)=\frac{x\times m_{ref}}{m}$$

The variance of this density function cannot be configured but varies depending on its expected value: the greater the distribution value expected (i.e., the average length of the microsatellite), the greater the variance of the distribution. This reliably reflects the behaviour of replication slippage errors, which increase in proportion to the length of the microsatellite sequence.

The mixture model is therefore defined for a vector of parameters θ by a density function $g_{\theta }\left(x\right)$, such as:

(A5) $$g_{\theta }\left(x\right)=f\left(x,m_{1}\right)\times P_{1}+f\left(x,m_{2}\right)\times P_{2}+f\left(x,m_{3}\right)\times \left(1-P_{1}-P_{2}\right)$$

### Appendix A.3. Expectation-Maximisation Algorithm

MEM uses the Expectation-Maximisation (EM) algorithm to determine which parameters, namely *m_1_*, *m_2_*, *m_3_*, *P_1_* and *P_2_*_,_ are used by the mixture model to represent the observed distribution with maximum likelihood.

Briefly, an initial value is assigned to each parameter ($m_{1}=m_{ref}+2$; $m_{2}=m_{ref}-3$; $m_{3}=m_{ref}-10$; $P_{1}=33\%$; $P_{2}=33\%$). The EM algorithm then operates according to two steps: at the expectation step, the algorithm determines, for each length i of the microsatellite, the extent to which the probability of occurrence $p_{i}$ can be explained by each sub-distribution *j*, by calculating a weight $w_{i,j}$ such that:

(A6) $$w_{i,j}=\frac{f\left(i,m_{j}\right)\times P_{j}}{g\left(i\right)}$$

At the maximisation step, the algorithm recalculates the value of the parameters from the observed data, weighted by $w_{i,j}$. Thus, the new value of the expected value $m_{j}$ of the sub-distribution j is:

(A7) $$m_{j}=\overset{L_{max}}{\underset{i=0}{\sum }}i\times p_{i}\times w_{i,j}$$

The new value of the proportion $P_{j}$ of the sub-distribution j in the mixture model is:

(A8) $$P_{j}=\overset{L_{max}}{\underset{i=0}{\sum }}p_{i}\times w_{i,j}$$

After the maximisation step, MEM calculates the log-likelihood $L_{g}\left(\theta \right)$ of the mixture model defined by the vector θ of the recalculated parameters $m_{1}$, $m_{2}$, $m_{3}$, $P_{1}$ and $P_{2}$:

(A9) $$\log L_{g}\left(\theta \right)=\overset{L_{max}}{\underset{i=0}{\sum }}\left(\log g_{\theta }\left(i\right)\times N_{i}\right)$$

Both steps are then repeated: with each repetition, the likelihood of the mixture model increases and tends asymptotically towards a maximum. The repetition of the EM algorithm is stopped when the log-likelihood stabilises or after 100 repetitions. The parameters $m_{1}$, $m_{2}$, $m_{3}$, $P_{1}$ and $P_{2}$, as well as the log-likelihood value obtained at the last repetition, are retained and define the optimal mixture model.

### Appendix A.4. Interpretation

If the expected value of a sub-distribution falls within an interval $\left[m_{ref}\times 90\%;m_{ref}\times 110\%\right]$ (i.e., if the expected value of the sub-distribution is equal to the mean of the reference distribution, +/−10%), then this sub-distribution is deemed to be related to a stable allele. Otherwise, the sub-distribution is deemed related to a potentially unstable allele. If no sub-distribution is associated with a possibly unstable allele, or if the sub-distributions associated with instability represent less than 2% of the mixture model, then the microsatellite is considered stable.

If there are one or more unstable sub-distributions representing more than 2% of the mixture model, then MEM defines a second mixture model q(x), including only stable sub-distributions:

(A10) $$q_{\theta }\left(x\right)=\frac{f\left(x,m_{1}\right)\times P_{1}\times E_{1}+f\left(x,m_{2}\right)\times P_{2}\times E_{2}+f\left(x,m_{3}\right)\times \left(1-P_{1}-P_{2}\right)\times E_{3}}{P_{1}\times E_{1}+P_{2}\times E_{2}+\left(1-P_{1}-P_{2}\right)\times E_{3}}$$

whereby $E_{j}$ is an indicator function equal to one if the sub-distribution j is considered stable, and equal to zero if the sub-distribution j is associated with instability. The log-likelihood $L_{q}\left(\theta \right)$ of this new mixture model is then calculated:

(A11) $$\log L_{q}\left(\theta \right)=\overset{L_{max}}{\underset{i=0}{\sum }}\left(\log q_{\theta }\left(i\right)\times N_{i}\right)$$

MEM finally evaluates the ability of the complete mixture model to represent the observed length distribution, compared to this new mixture model q(x), including only the stable sub-distributions, using the log-likelihood ratio test. The statistic λ is:

(A12) $$\lambda =-2\left[\log L_{q}\left(\theta \right)-\log L_{g}\left(\theta \right)\right]$$

A cut-off value of 4 was defined in a development cohort of 36 MSS patients with 5 stable microsatellites and 15 MSI patients with 5 unstable microsatellites in MSI-PCR. If the log-likelihood ratio is greater than four, then the complete mixture model represents the observed length distribution to a significantly better extent than the mixture model, including only the stable sub-distributions: the microsatellite is deemed unstable, and MEM can estimate the allele frequency and the length of unstable alleles from the parameters of the mixture model.

Otherwise, it is necessary to determine whether the number of sequences obtained for the microsatellite is sufficient to detect an unstable allele. The statistical power of the log-likelihood ratio test according to the number of microsatellite sequences was determined in advance for each microsatellite, using simulated data. In the case of a negative log-likelihood ratio test, the microsatellite analysis is considered to be contributory only if the number of sequences is sufficient to obtain a statistical power greater than 80% to detect an unstable allele present at a frequency of 10%, and with a length equal to $m_{ref}\times 90\%-1$. If so, the microsatellite is deemed stable; otherwise, the analysis of this microsatellite is considered non-contributory.

Similar to MSI-PCR, a sample is considered as MSI if at least two microsatellites out of five are unstable. The sample is considered MSS if at least four microsatellites out of five are stable. The sample analysis is deemed non-contributory if at least two microsatellites are non-contributory or if at least one microsatellite is non-contributory and another is unstable.

MEM source code and ready-to-use files for CLC Genomics Workbench are available at https://github.com/MGPC-Nantes/MEM (accessed on 19 August 2021).
