# Supplementary material for: MEM: An Algorithm for the Reliable Detection of Microsatellite Instability (MSI) on a Small NGS Panel in Colorectal Cancer
Source: Cancers (Basel). 2021 Aug 20;13(16):4203. doi: 10.3390/cancers13164203 (PMC8394433; doi:10.3390/cancers13164203)
Supplement: Supplementary file 1 [file cancers-13-04203-s001.zip › cancers-1318212-supplementary.pdf]

cancers

MDPI

Supplementary Table S1. Monomorphic microsatellites used by MEM for the determination of MSI status, genomic position of QIAseq AMP primers and MEM trimming parameters.

Name	Genomic position (hg38)	AMP primers genomic positions and strand (hg38)	Trimming parameters
**BAT-25**	chr4:54732046-54732070	chr4:54731989-54732023(+)	5′ trimmed sequence<br>CTCCAAGAATGTAAGTGGGAGTGATTCTCTAAAGAGTTTTTTGTGTTTTKTTTTTTTG<br>Mismatch cost = 2; Gap cost = 3; Minimum score = 45
			3′ trimmed sequence<br>AGAAMAGAGCATTTTAGA<br>Mismatch cost = 1; Gap cost = 1; Minimum score = 10
**BAT-26**	chr2:47414421-47414447	chr2:47414346-47414382(+)<br>chr2:47414366-47414398(+)<br>chr2:47414478-47414520(-)	5′ trimmed sequence<br>ACTGACTACTTTTGACTTCAGCCAGTATATGAAATTGGAWATTGCARMARWCAR…<br>AGCCCTTAAMCTTTTTYARG<br>Mismatch cost = 2; Gap cost = 3; Minimum score = 45
			3′ trimmed sequence<br>GGTTAAAAATGTTGAATGGTTAAAAAAGTTTT<br>Mismatch cost = 1; Gap cost = 1; Minimum score = 12
**NR-21**	chr14:23183138-23183158	chr14:23183089-23183116(+)	5′ trimmed sequence<br>CATTCACACTTTCTGGTCACTCGCGTTTACAAAMAARAAAARTGTT<br>Mismatch cost = 2; Gap cost = 3; Minimum score = 20
			3′ trimmed sequence<br>GCCAGGGGRGACAWAMATTT<br>Mismatch cost = 1; Gap cost = 1; Minimum score = 8
**NR-24**	chr2:95183614-95183636	chr2:95183559-95183591(+)	5′ trimmed sequence<br>GCTGAATTTTACCTCCTGACTCCAAAAACTCTTCTCTTCCC<br>TGGGCCCAGTC<br>Mismatch cost = 2; Gap cost = 3; Minimum score = 25
			3′ trimmed sequence<br>TGAGACARAGTCTCMCTCTK<br>Mismatch cost = 1; Gap cost = 1; Minimum score = 10
**NR-27**	chr2:39309549-39309575	chr2:39309446-39309481(+)	5′ trimmed sequence<br>GGCTATACTACTTACAAGTTCATGATGTGGTTCTGTCTCCTTTCTTAAGGGTGGAT…<br>CAAATTTCACTTGGCCAACTAAAAAARAAAAAAAAGTAAAACCA<br>Mismatch cost = 2; Gap cost = 3; Minimum score = 30
			3′ trimmed sequence<br>RGGCAGAGTYTTGCTCTGTCTCCC<br>Mismatch cost = 1; Gap cost = 1; Minimum score = 8
